# Dose delay, dose reduction, and early treatment discontinuation in Black and White women receiving chemotherapy for nonmetastatic breast cancer

**DOI:** 10.1093/oncolo/oyae150

**Published:** 2024-06-24

**Authors:** Moriah Forster, Allison M Deal, Annie Page, Sanah Vohra, Alexis C Wardell, Joyce Pak, Jennifer L Lund, Kirsten A Nyrop, Hyman B Muss

**Affiliations:** Division of Hematology and Oncology, Department of Medicine, Vanderbilt University, Nashville, TN 37232, United States; Lineberger Comprehensive Cancer Center, University of North Carolina, Chapel Hill, NC 27599, United States; Department of Epidemiology, Gillings School of Global Public Health, University of North Carolina, Chapel Hill, NC 27599, United States; Lineberger Comprehensive Cancer Center, University of North Carolina, Chapel Hill, NC 27599, United States; Department of Epidemiology, Gillings School of Global Public Health, University of North Carolina, Chapel Hill, NC 27599, United States; Lineberger Comprehensive Cancer Center, University of North Carolina, Chapel Hill, NC 27599, United States; Department of Epidemiology, Gillings School of Global Public Health, University of North Carolina, Chapel Hill, NC 27599, United States; Lineberger Comprehensive Cancer Center, University of North Carolina, Chapel Hill, NC 27599, United States; Department of Epidemiology, Gillings School of Global Public Health, University of North Carolina, Chapel Hill, NC 27599, United States; Lineberger Comprehensive Cancer Center, University of North Carolina, Chapel Hill, NC 27599, United States; Department of Medicine, School of Medicine, University of North Carolina, Chapel Hill, NC 27599, United States; Lineberger Comprehensive Cancer Center, University of North Carolina, Chapel Hill, NC 27599, United States; Department of Medicine, School of Medicine, University of North Carolina, Chapel Hill, NC 27599, United States

**Keywords:** breast cancer, chemotherapy, race, toxicity

## Abstract

**Background:**

To describe reasons for deviations from planned chemotherapy treatments in women with nonmetastatic breast cancer that contribute to less-than-planned receipt of chemotherapy.

**Methods:**

Electronic medical records for patients receiving chemotherapy were reviewed for adverse events and treatment modifications. Log-binomial regression models were used to estimate relative risks (RRs) with 95% CIs to examine associations between chemotherapy modifications, patient characteristics, and treatment modalities.

**Results:**

Delays in chemotherapy initiation (7%) were for surgical complications (58%), personal reasons (16%), and other (26%; port malfunction, infections, and obtaining extra imaging). Delays during chemotherapy (38%) were for infections (20%), neutropenia (13%), and personal reasons (13%). Dose reductions (38%) were for neuropathy (36%), unknown causes (9%), anemia (9%), and neutropenia (8%). Early treatment discontinuations (23%) were for neuropathy (29%). Patients receiving paclitaxel/nab-paclitaxel (RR 2.05; 95% CI, 1.47-2.87) and an anthracycline (RR 1.89; 95% CI, 1.39-2.57) reported more dose delays during chemotherapy. Black race (RR 1.46; 95% CI, 1.07-2.00), stage 3 (RR 1.79; 95% CI, 1.09-2.93), and paclitaxel/nab-paclitaxel receipt (RR 1.39; 95% CI, 1.02-1.90) increased the likelihood of dose reduction. Both Black race (RR 2.06; 95% CI, 1.35-3.15) and receipt of paclitaxel/nab-paclitaxel (RR 1.93; 95% CI, 1.19-3.13) increased the likelihood of early discontinuation. Patients receiving anthracyclines had higher rates of hospitalizations during chemotherapy (RR: 1.79; 95% CI, 1.11-2.89).

**Conclusion:**

Toxicities are the most common reason for treatment modifications and need close monitoring in high-risk groups for timely intervention. Dose reductions and early treatment discontinuations occurred more for Black patients and need further study.

## Teaser

This article describes reasons for deviations from planned chemotherapy treatments in women with nonmetastatic breast cancer, considering those that contribute to less-than-planned receipt of chemotherapy.

**Implications for Practice**

Most reasons for chemotherapy modifications in patients with early-stage breast cancer were for treatment-related toxicities. This highlights the need for symptom-focused care and early intervention to enable patients to receive and complete their chemotherapy on schedule.

## Introduction

A combination of surgery, radiation, chemotherapy, and/or endocrine therapy is the mainstay for curative treatment of nonmetastatic breast cancer (BC).^1^ The 10-year mortality risk from BC can be reduced by one-third by the inclusion of chemotherapy in patients receiving this treatment on a standard-of-care dosing schedule.^2^ However, modifications to chemotherapy dosing and schedule are common. For example, in a study of 20 799 women with early-stage BC receiving care at community oncology clinics, a chemotherapy dose reduction occurred in 37% of patients and a chemotherapy delay by 7 or more days occurred in 25% of patients.^3^ Delays in chemotherapy initiation^1,4-8^ and reductions in chemotherapy dose^9^ can worsen survival and increase risk of death.^1,10^

Several studies have assessed chemotherapy modifications and their associations with patient or tumor characteristics.^4,5,7,9,11^ Black women and older patients have been identified as at higher risk for chemotherapy delays, dose reductions, and early discontinuations as compared to their White and younger counterparts.^4-6,8,9,11-17^ It is important to understand why there are deviations to the planned chemotherapy regimen given these observed disparities. A deeper understanding of the reasons behind chemotherapy modifications can be informative for the development of timely interventions that may improve treatment adherence and thereby reduce disparities in chemotherapy receipt and survival.

We performed a retrospective chart review of women with nonmetastatic BC receiving chemotherapy to further elucidate reasons for chemotherapy delays, reductions, and discontinuations. We investigated associations between treatment modifications and patient characteristics, BC diagnosis, surgery, specific chemotherapy regimens (anthracycline vs nonanthracycline and paclitaxel vs docetaxel), and chemotherapy timing (adjuvant vs neoadjuvant). We also evaluated the longitudinal cumulative dose (LCD) by race and age of the patient to better describe the impacts of chemotherapy dose modifications over time. The overall objective was to identify patients who may be at the highest risk for unplanned modifications in their chemotherapy treatment schedule and thereby warrant close monitoring for early intervention opportunities.

## Materials and methods

### Study participants

The creation of a database of women with nonmetastatic BC (stages 1-3) was approved by the University of North Carolina’s (UNC) Institutional Review Board as described previously.^18-20^ Patients treated within UNC Health clinics outside of a clinical trial were identified through reviews of the daily clinic schedule and received chemotherapy between March 2014 and March 2020.

The electronic medical record (EMR) was reviewed to extract demographic information, BC stage and phenotype, dates of BC diagnosis and surgery, chemotherapy regimen, timing and dosing of chemotherapy, and hospitalization during chemotherapy. All data were collected and managed using Research Electronic Data Capture (REDCap) tools hosted by the UNC School of Medicine.^21,22^

### Chemotherapy treatment

Each chemotherapy infusion record was entered into the REDCap database, including medication name, dose, and date of infusion. We compared this information to clinician notes where the intended treatment plan was described to identify any deviations in the course of chemotherapy. If the schedule of the chemotherapy regimen was not clear in the clinician notes, we referred to UpToDate^23^ for the recommended timing and dose of common chemotherapy regimens (Table A1). In this manner, we identified delays in chemotherapy, dose reductions, or early discontinuation of individual chemotherapy drugs, and reasons for treatment modifications as indicated in clinician notes. A delay in chemotherapy initiation and a delay during chemotherapy was defined as the chemotherapy being given 6 or more days later than indicated in the initial treatment plan or as recommended in UpToDate.^23^ A dose reduction was defined as a deviation from the planned total dosage that occurred at any time during the treatment. A dose reduction was recorded if even one of the chemotherapy agents was reduced. Early treatment discontinuation was documented if all chemotherapy was discontinued.

Moreover, the LCD was calculated for each drug and the corresponding dosing schedule for a subsample of the population for which we had data (*N* = 207). Patients who did not have drug-level data available (*N* = 28) and those who had an unconventional chemotherapy regimen (*N* = 35) were excluded. LCD is defined at any given time for a given agent as the sum of all doses received up to and including that time point divided by the standard dose that a patient should have received at the end of their treatment.^24^ The LCD at the final time point for any given agent and corresponding dosing schedule is equivalent to the relative dose intensity (RDI). The RDI for any given agent is defined as the total dose the patient received during their treatment regimen divided by the standard dose the patient should have received at the end of the treatment regimen. RDI was calculated for all drugs except carboplatin. We created a regimen-level summary variable indicating if RDI was <85% for at least one agent in the patients’ respective regimen.

### Statistical considerations

Descriptive statistics were calculated to summarize patient characteristics, BC tumor characteristics, treatment type and timing, chemotherapy regimen, and reasons for modifications overall and by race. Associations with study variables and race were evaluated using Fisher’s exact tests for categorical variables and Kruskal-Wallis Tests for continuous variables. Log-binomial regression models were used to estimate relative risks (RRs) and 95% CIs as a measure of association between chemotherapy modifications (initiation delay, dose delay, dose reduction, early discontinuation), hospitalizations, and RDI <85% with patient characteristics, BC stage, and treatment type/timing. Multivariable adjusted analyses were limited to variables significant in unadjusted analysis. Stage, chemotherapy timing, receipt of anthracycline, and taxane type had significant multicollinearity and were therefore not included together in any adjusted analyses. All analyses were conducted in SAS version 9.4 (SAS Institute, Cary, NC). Statistical significance was defined as *P* ≤ .05.

## Results

### Sample characteristics

Our analysis included 270 patients which included 62 Black patients (23%) and 208 White patients (77%; Table 1). The average age at BC diagnosis was 56 years old (range 23-83 years). Most patients received adjuvant chemotherapy (63%). Forty-one percent received an anthracycline-based regimen. Most regimens (97%) included a taxane. All patients received supportive care (including growth factor if indicated) as per standard of care. The only significant difference in sample characteristics between Black and White patients was BC subtype, with triple-negative tumors more prevalent in Black (37%) versus White patients (24%; *P* = .02).

**Table 1.** Study participant characteristics at BC diagnosis.

Variable	*N* = 270	White	Black	*P*-value
Age at diagnosis—mean (range)	56 (23-83)			
<65	176 (65%)	130 (63%)	46 (74%)	0.10
≥65	94 (35%)	78 (37%)	16 (26%)	
Race				
White	208 (77%)	208	62	N/A
Black	62 (23%)			
Stage				
1	61 (22%)	46 (22%)	15 (24%)	.93
2	145 (54%)	113 (54%)	32 (52%)	
3	64 (24%)	49 (24%)	15 (24%)	
Subtype				
HR−/HER2− (triple-negative)	73 (27%)	50 (24%)	23 (37%)	.02^a^
HR−/HER2+	27 (10%)	18 (9%)	9 (14%)	
HR+/HER2−	127 (47%)	108 (52%)	19 (31%)	
HR+/HER2+	43 (16%)	32 (15%)	11 (18%)	
Chemotherapy timing				
Neoadjuvant	100 (37%)	78 (37%)	22 (35%)	.89
Adjuvant	170 (63%)	130 (63%)	40 (65%)	
Anthracycline-based regimen				
No	159 (59%)	121 (58%)	38 (61%)	.77
Yes	111 (41%)	87 (42%)	24 (39%)	
Taxane-based regimen				
None	9 (3%)	6 (3%)	3 (5%)	.43
Paclitaxel/nab-Paclitaxel	119 (44%)	92 (44%)	27 (44%)	
Docetaxel	138 (51%)	108 (52%)	30 (48%)	
Both	4 (2%)	2 (1%)	2 (3%)	
Chemotherapy regimen				
Doxorubicin/cyclophosphamide before/after paclitaxel (AC-T)	80 (30%)	60 (29%)	20 (32%)	.08
Doxorubicin/cyclophosphamide before/after paclitaxel/carboplatin (AC-TC)	21 (8%)	19 (9%)	2 (3%)	
Docetaxel/cyclophosphamide (± anti-HER-2; TC)	87 (32%)	73 (35%)	14 (23%)	
Docetaxel/carboplatin/anti-HER-2 (TCH)	44 (16%)	31 (15%)	13 (21%)	
Other	38 (14%)	25 (12%)	13 (21%)	

^a^Statistically significant *P*-value.

### Chemotherapy regimen deviations

In Table 2, we show reasons for dose modifications by race. In Table 3, we show unadjusted associations with chemotherapy modifications.

**Table 2.** Reasons for dose modifications by race.

	Total, *N* (%)	White, *N* (%)	Black, *N* (%)
Dose delay—initiation^a^			
No	251 (93%)	194 (93%)	57 (92%)
Yes	19 (7%)	14 (7%)	5 (8%)
Primary reasons for dose delay—initiation (*N* = 19)			
Surgical complications	11 (58%)	9 (64%)	2 (40%)
Personal reasons	3 (16%)	2 (14%)	1 (20%)
Other	5 (26%)	3 (21%)	2 (40%)
Dose delay—during chemotherapy^b^			
No	168 (62%)	135 (65%)	33 (53%)
Yes	102 (38%)	73 (35%)	29 (47%)
Primary reasons for dose delay after first chemotherapy dose (*N* = 105)			
Other	22 (22%)	15 (21%)	7 (24%)
Infections and/or Sepsis	20 (20%)	16 (22%)	4 (14%)
Neutropenia (without fever)	14 (13%)	10 (14%)	4 (14%)
Unknown	14 (13%)	12 (16%)	2 (7%)
Personal reasons	13 (13%)	9 (12%)	4 (14%)
Neuropathy	12 (12%)	9 (12%)	3 (10%)
Anemia	7 (7%)	2 (3%)	5 (17%)
Dose reduction^c^			
No	168 (62%)	137 (66%)	31 (50%)
Yes	102 (38%)	71 (34%)	31 (50%)
Primary reasons for dose reduction (*N* = 102)			
Neuropathy	37 (36%)	27 (38%)	10 (33%)
Other	13 (13%)	10 (14%)	3 (10%)
Unknown	10 (9%)	9 (13%)	1 (3%)
Anemia	9 (9%)	3 (4%)	6 (20%)
Neutropenia (without fever)	8 (8%)	5 (7%)	3 (10%)
Fatigue	6 (6%)	4 (6%)	2 (6%)
Thrombocytopenia	5 (5%)	3 (4%)	2 (6%)
Nausea/vomiting	5 (5%)	3 (4%)	2 (6%)
Neutropenic fever	5 (5%)	4 (6%)	1 (3%)
Infections and/or sepsis	4 (4%)	3 (4%)	1 (3%)
Early treatment discontinuation^d^			
No	207 (77%)	169 (81%)	38 (61%)
Yes	63 (23%)	39 (19%)	24 (39%)
Primary reasons for early treatment discontinuation (*N* = 63)			
Other	24 (38%)	15 (38%)	9 (38%)
Neuropathy	18 (29%)	10 (26%)	8 (33%)
Infusion reaction	6 (9%)	4 (10%)	2 (8%)
Unknown	6 (9%)	6 (16%)	0 (0%)
Personal reasons	5 (9%)	4 (10%)	1 (4%)
Infections and/or sepsis	4 (6%)	0 (0%)	4 (17%)
Hospitalization			
No	216 (80%)	170 (81%)	46 (74%)
Yes	54 (20%)	38 (19%)	16 (26%)
Primary reasons for hospitalization (*N* = 54)			
Other	20 (37%)	13 (34%)	7 (43%)
Neutropenic fever	19 (35%)	13 (34%)	6 (38%)
Infections and/or sepsis	15 (28%)	12 (32%)	3 (19%)

^a^Dose delay in chemotherapy initiation was defined as 6 or more days after the proposed initiation date.

^b^Deviation from the planned infusion intervals 6 or more days after the regularly scheduled date.

^c^Deviation from the planned total dosage that occurred at any time during the treatment.

^d^Deviation from the planned number of chemotherapy infusions.

**Table 3.** Unadjusted analysis of predictors with chemotherapy modifications.

Predictor	Total *N*	Chemotherapy initiation delay		Dose delay during chemotherapy		Dose reduction		Early discontinuation		Hospitalization	
		*N* (%)	RR (95% CI)	*N* (%)	RR (95% CI)	*N* (%)	RR (95% CI)	*N* (%)	RR (95% CI)	*N* (%)	RR (95% CI)
Age											
<65	176	13 (7%)	1.00	72 (41%)	1.00	67 (38%)	1.00	40 (23%)	1.00	39 (22%)	1.00
≥65	94	6 (6%)	0.86 (0.34-2.20)	30 (32%)	0.78 (0.55-1.10)	35 (37%)	0.98 (0.71-1.35)	23 (24%)	1.08 (0.69-1.68)	15 (16%)	0.72 (0.42-1.24)
Race											
White	208	14 (7%)	1.00	73 (35%)	1.00	71 (34%)	1.00	39 (19%)	1.00	38 (18%)	1.00
Black	62	5 (8%)	1.20 (0.45-3.20)	29 (47%)	1.33 (0.96-1.84)	31 (50%)	1.46 (1.07-2.00)^a^	24 (39%)	2.06 (1.35-3.15)^a^	16 (26%)	1.41 (0.85-2.35)
Stage											
1	61	6 (10%)	1.00	19 (31%)	1.00	16 (26%)	1.00	17 (28%)	1.00	7 (11%)	1.00
2	145	9 (6%)	0.63 (0.23-1.70)	53 (37%)	1.17 (0.76-1.80)	56 (39%)	1.47 (0.92-2.35)	27 (19%)	0.67 (0.39-1.13)	31 (21%)	1.86 (0.87-4.00)
3	64	4 (6%)	0.64 (0.19-2.14)	30 (47%)	1.50 (0.95-2.37)	30 (47%)	1.79 (1.09-2.93)^a^	19 (30%)	1.07 (0.61-1.85)	16 (25%)	2.18 (0.96-4.93)
Chemotherapy timing											
Neoadjuvant	100	1 (1%)	N/A^b^	45 (45%)	1.00	50 (50%)	1.00	27 (27%)	1.00	20 (20%)	1.00
Adjuvant	170	18 (11%)	N/A^b^	57 (34%)	0.75 (0.55-1.01)	52 (31%)	0.61 (0.45-0.83)^a^	36 (21%)	0.78 (0.51-1.21)	34 (20%)	1.00 (0.61-1.64)
Anthracycline-based regimen											
No	159	15 (9%)	1.00	44 (28%)	1.00	56 (35%)	1.00	31 (20%)	1.00	24 (15%)	1.00
Yes	111	4 (4%)	0.38 (0.13-1.12)	58 (52%)	1.89 (1.39-2.57)^a^	46 (41%)	1.18 (0.87-1.60)	32 (29%)	1.48 (0.96-2.27)	30 (27%)	1.79 (1.11-2.89)^a^
Taxane											
Docetaxel	138	12 (9%)	1.00	35 (25%)	1.00	45 (33%)	1.00	21 (15%)	1.0	22 (16%)	1.00
Paclitaxel/nab-paclitaxel	119	6 (5%)	0.58 (0.22-1.50)	62 (52%)	2.05 (1.47-2.87)^a^	54 (45%)	1.39 (1.02-1.90)^a^	35 (29%)	1.93 (1.19-3.13)^a^	27 (23%)	1.42 (0.86-2.36)

^a^Statistically significant *P*-value.

^b^ *N* was too small to calculate the RR.

#### Chemotherapy initiation

Nineteen patients (7%) had a delay in chemotherapy initiation for reasons including surgical complications (58%), personal reasons (16%; obtaining a second opinion, relocating, and arranging personal affairs before chemotherapy), and other (26%; port malfunction, infections, and obtaining extra imaging; Table 2). No differences were seen by age, race, disease, or treatment characteristics (Table 3).

#### Chemotherapy delays during treatment

One hundred and two patients (38%) had at least one delay during chemotherapy for reasons including infections (20%), neutropenia (13%), personal reasons (13%; leaving the infusion clinic due to long wait, transportation barriers, and vacations), unknown causes (13%), neuropathy (12%), and anemia (7%; Table 2). Anthracycline and paclitaxel/nab-paclitaxel were associated with an increased likelihood of experiencing dose delay during chemotherapy (RR 1.89; 95% CI, 1.39-2.57; *P* ≤ .01 and RR 2.05; 95% CI, 1.47-2.87; *P* ≤ .01; Table 3). Black patients receiving “other” chemotherapy regimens (Table A2) were more likely to have a delay during chemotherapy (85% vs 36%).

#### Dose reductions

One hundred and two patients (38%) experienced at least one dose reduction for reasons including neuropathy (36%), unknown causes (9%), anemia (9%), neutropenia (8%) fatigue (6%), and thrombocytopenia (5%; Table 2). Black patients were more likely than White to experience a dose reduction (RR 1.46; 95% CI, 1.07-2.00; *P* = .02), as were patients with stage 3 as compared to stage 1 (RR 1.75; 95% CI, 1.09-2.93; *P* = .02). Adjuvant chemotherapy decreased the likelihood of dose reduction compared to neoadjuvant (RR 0.61; 95% CI, 0.45-0.83; *P* = .01). Paclitaxel/nab-paclitaxel increased the likelihood of dose reduction (RR 1.39; 95% CI, 1.02-1.90; *P* = .04) as compared to docetaxel (Table 3). In a multivariable model including both race and stage, 2 variables maintained their significant relationship with dose reductions: Black vs White adjusted RR 1.47; 95% CI, 1.09-2.00; *P* = .01, and stage 3 vs stage 1 adjusted RR 1.79; 95% CI, 1.10-2.92; *P* = .02. Black patients receiving docetaxel, carboplatin, and trastuzumab (TCH) were more likely to experience a dose reduction than White patients (92% vs 52%; Table A2).

#### Early treatment discontinuation

Sixty-three patients (23%) had early treatment discontinuation for reasons including neuropathy (29%), personal (9%; relocating, getting treatment elsewhere, and no longer wanting chemotherapy), infusion reaction (9%), unknown causes (9%), and infections (6%; Table 2). Black patients were twice as likely to experience early discontinuation as compared to White patients (RR 2.06; 95% CI, 1.35-3.15; *P* = .01). Receipt of paclitaxel/nab-paclitaxel was significantly associated with early discontinuation (RR 1.93; 95% CI, 1.19-3.13; *P* = .01; Table 3). In a multivariable model including both race and taxane receipt, both variables maintained their significant association with early discontinuation: Black versus White adjusted RR 1.78; 95% CI, 1.12-2.81; *P* = .01 and paclitaxel/nab-paclitaxel versus docetaxel adjusted RR 1.92; 95% CI, 1.19-3.09; *P* = .01. Black patients receiving doxorubicin, cyclophosphamide, and paclitaxel (AC-T) and “other” chemotherapy regimens were more likely to experience early treatment discontinuation than White patients (45% vs 17% and 77% vs 40%, respectively; Table A2).

#### Hospitalizations

Fifty-four patients (20%) experienced hospitalization during chemotherapy for reasons including neutropenic fever (35%) and infections (28%; Table 2). Patients receiving an anthracycline (RR 1.79; 95% CI, 1.11-2.89; *P* = .02) were more likely to be hospitalized as compared to a nonanthracycline regimen (Table 3).

#### RDI <85% at the end of treatment

**Figure A1.**
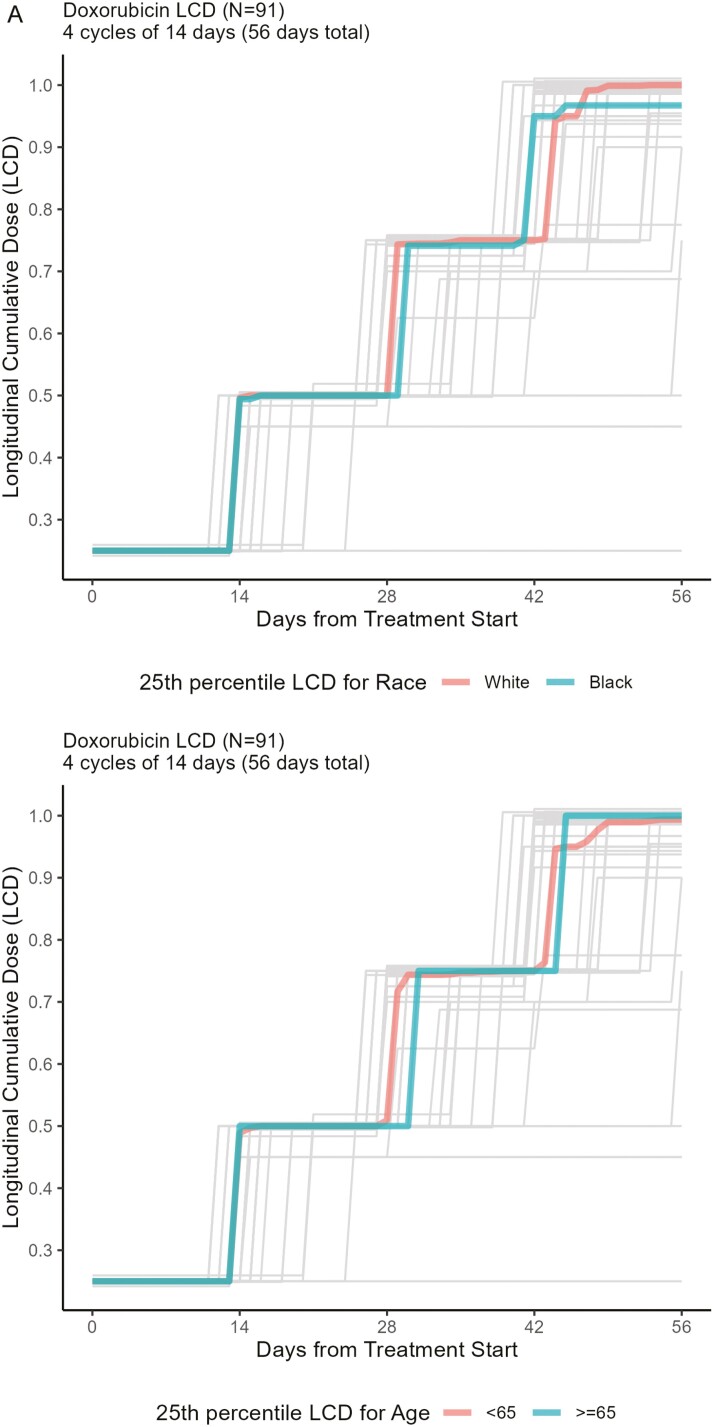

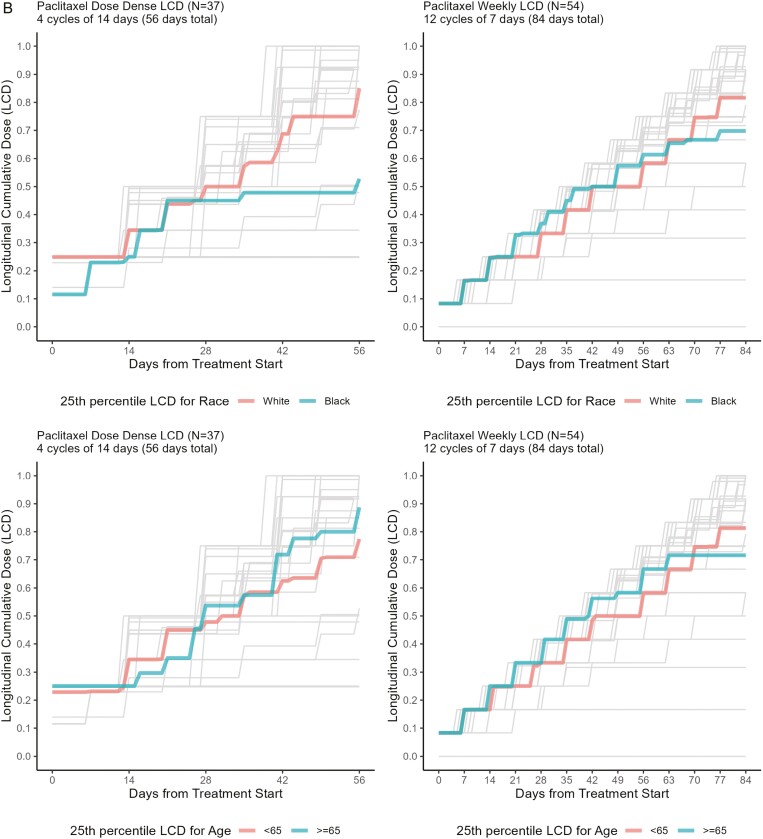

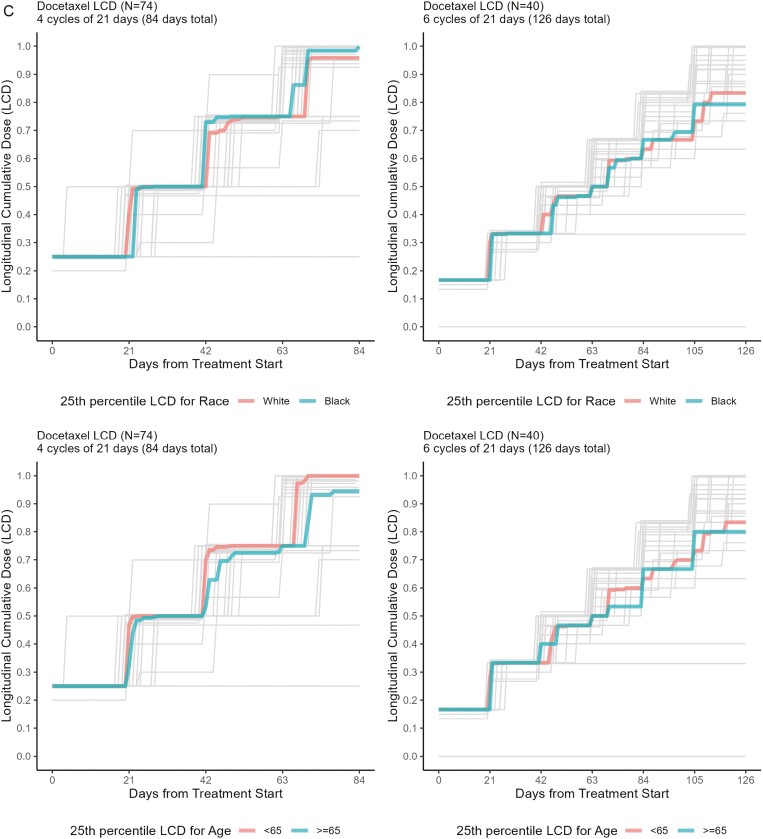

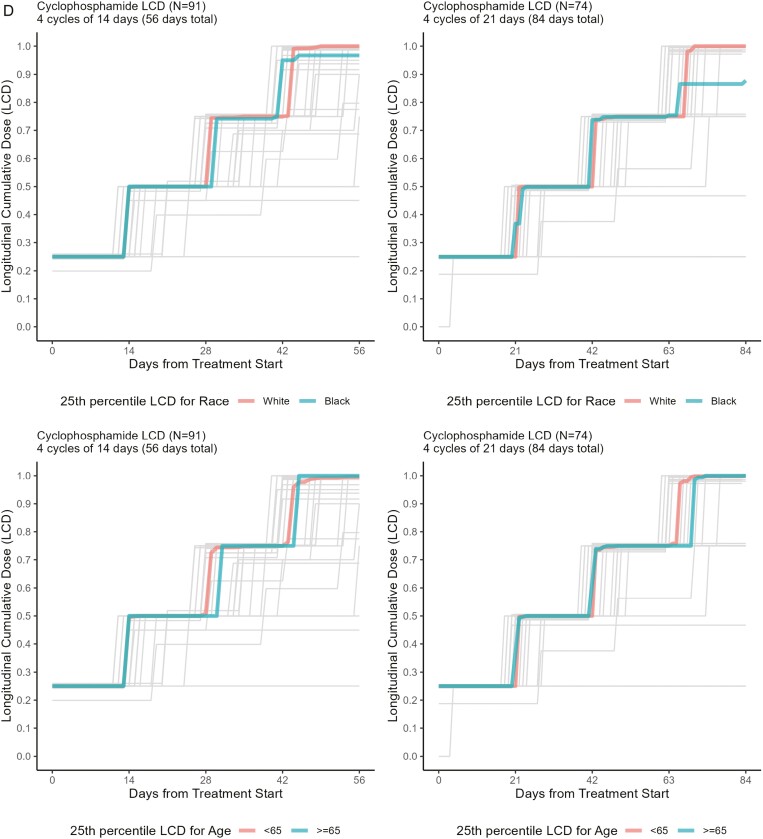
Longitudinal cumulative dose (LCD) plots for the agent and dosing schedules. In each panel, light gray lines represent the LCD of each person who received the agent and the respective dosing schedule. In Panel A (Doxorubicin), the 25th percentile LCD for both race and age groups are depicted by the colored lines. In Panel B (Paclitaxel), there are 2 different dosing schedules, each dosing schedule is represented by a column in the panel. The 25th percentile LCD for both race and age groups is depicted by the colored lines. Panel C (Docetaxel) and Panel D (Cyclophosphamide) are similar to Panel B. Only the 25th percentile is depicted in all Panels because, for the majority of agents, a large proportion of the patient population received most if not all of the standard dose, thus making the 50th and 75th percentiles visually indistinguishable. The exception is dose-dense paclitaxel where Black patients have lower LCD.

In an exploratory analysis of a subpopulation of 207 patients, 56 patients (27%) had an RDI <85% for at least one of the agents in their treatment regimen. There were no statistically significant associations between RDI being <85% at the end of treatment and age, race, diagnosis stage, the timing of chemotherapy, and receipt of an anthracycline (Table A3). In this subsample, the proportion of Black patients who had an RDI <85% for at least one of the agents in their regimen was 36% in comparison to 25% of White patients (RR: 1.48; 95% CI, 0.92-2.38; *P* = .10). LCD plots for each agent/dose time combination are included in Figure A1. The LCD plots suggest that for most drugs there are no large differences by age or race. However, for those receiving dose-dense paclitaxel, Black patients appear to have lower LCD.

## Discussion

The aim of this study was to elucidate the reasons behind chemotherapy modifications in patients with early-stage BC. Understanding these reasons is essential to effective monitoring of patients at the highest risk for treatment modifications and offering timely interventions to help them complete their full chemotherapy course. We explored specific reasons for chemotherapy modifications and conducted multivariable analyses to identify independently significant factors.

### Clinical reasons

Delays in chemotherapy initiation were primarily for surgical complications. Delays during chemotherapy were caused by many known toxicities (infections/sepsis, neutropenia, and neuropathy), and dose reductions and early discontinuations were mostly due to neuropathy. Receipt of an anthracycline was associated with a higher risk for a delay during therapy and hospitalization, most likely due to the higher toxicity profile of these regimens as compared to nonanthracycline-based regimens.^25^ Patients receiving paclitaxel/nab-paclitaxel were also more likely to have a dose delay, dose reduction, and early discontinuation as compared to docetaxel, largely due to greater odds of developing peripheral neuropathy.^26^

Other studies have identified reasons for chemotherapy modifications. In a study of women receiving adjuvant anthracycline-based therapy, reasons for modification were neutropenia, unspecified personal reasons, thrombocytopenia, and anemia.^10^ Another study assessed dose reductions or delays in patients receiving neoadjuvant chemotherapy and reported neuropathy, fatigue, and myelosuppression.^27^ In a third study, reasons for dose delays included acute illness, hospitalization, neutropenia, leukopenia, missed appointments, and surgical complications.^28^ In our sample, clinical reasons for chemotherapy modifications were largely similar between Black and White patients (Table 2).

### Personal reasons

In our sample, personal reasons for treatment modifications included seeking a second opinion, travel, moving, transportation barriers, and personal decision-making. Other studies have similarly reported delays in chemotherapy due to seeking a second opinion, missed appointments, scheduling issues, family events, patient decision-making, losing employment due to the diagnosis, and operating room availability.^11,15,29^ In the Carolina Breast Cancer Study, a delay in treatment from the time of diagnosis was associated with smaller household size, losing a job due to the BC diagnosis, and breast reconstruction immediately after mastectomy.^11^ Another study reported that patients who experience more cancer-related distress have a lower likelihood of completing 85% of the prescribed chemotherapy on time.^9^

### Delays in chemotherapy treatment

Our study adds to previous research regarding delays in chemotherapy initiation,^4,5,7,8,11,12,15^ especially the exploration of reasons for during-chemotherapy treatment delays, which have not been investigated as extensively. One study investigated during-chemotherapy delays in older patients with colorectal, breast, and lung cancer treated between 1998 and 2008 and reported that 22% of delays were at the patient’s request.^30^ Other studies have focused on the total duration of treatment^14^ or the ratio of the actual duration to the expected duration of treatment^28^ as a surrogate for during-chemotherapy delays. Our study adds to the literature by exploring the reasons for these delays.

### Race disparity

Our study also adds to the literature with regard to the potential racial disparity in chemotherapy modifications. Black women are more likely to be diagnosed with triple-negative BC, so they accordingly receive longer, more intensive chemotherapy regimens (like AC-T). We saw this disparity in the BC stage in our sample. Furthermore, Black patients in our sample experienced more dose delays during chemotherapy when receiving “other” chemotherapy regimens. They experienced more dose reductions, significantly more so when receiving TCH chemotherapy. They also experienced more early discontinuations, significantly more so when receiving AC-T or “other” chemotherapy regimens. We hypothesize that some of this disparity could be due to the more intensive chemotherapy regimens received for the more aggressive subtype of BC. Other studies have noted that even when controlling for insurance type, Black women experience more delays in their BC treatment.^31^ Black women have also been shown to experience a more prolonged treatment duration than White women, which suggests they experienced more during-chemotherapy treatment delays.^32^ In our sample, as noted earlier, the specific reasons for chemotherapy modifications were largely similar between Black and White patients. We conclude that the modifications that occur for Black patients are for similar reasons; however, they occur more frequently. This is an area for further research to better understand this disparity.

### Longitudinal cumulative dose

Our study is strengthened by the evaluation of LCD in a subgroup of the study population. LCD can be used to visualize the timing of chemotherapy deviations throughout treatment.^24^ When we looked at LCD for each chemotherapy drug based on the timing of the regimen (Figure A1), we noted that for dose-dense paclitaxel, Black patients have a lower LCD. This finding is supported by the fact that we already saw that Black patients in our study have higher rates of dose reductions as well as early discontinuations. This fits with other reported data where dose reductions in Black patients were seen more often with nonanthracycline-containing regimens.^33^

### Limitations

We relied on EMR documentation for dose modifications, which was not always consistently recorded in clinician notes. At times, more than one reason for modification was given, leaving the reviewer (MF) to choose the one that seemed most significant. Furthermore, we do not have granular detail on what chemotherapy modifications came during what aspect of chemotherapy (ie, during the anthracycline vs during the taxane). Also, this was a single-institution study at a university-affiliated hospital, which limits generalizability to other settings.

### Strengths

We reviewed clinician notes to understand the specific reasons behind chemotherapy modifications. This is important to understand why certain groups of patients are at higher risk and where toxicity monitoring should be focused for timely interventions that enable treatment adherence. Many studies have noted patient groups at risk for treatment delays and less than optimal treatment completion, but these data become more actionable when specific reasons are understood. Our study explores not only delays in the initiation of chemotherapy but also within-treatment delays. We also explore potential differences in adverse events by type of treatment regimen used in current clinical practice. The addition of LCD data helps visualize treatment disparities by race, age, and chemotherapy agent.

## Conclusion

The aim of our study was to identify the precise reasons for modifications to chemotherapy treatment plans among Black and White women treated with curative intent for nonmetastatic BC. We found that most reasons were for treatment-related toxicities, highlighting the need for symptom-focused care and early intervention to enable patients to receive and complete their chemotherapy on schedule.

## Data Availability

The data underlying this article will be shared on reasonable request to the corresponding author.

## Appendix

**Table A1.** Timing and dosing of 4 common BC chemotherapy regimens.

TC (docetaxel and cyclophosphamide)	
Docetaxel	IV 75 mg/m^2^
Cyclophosphamide	IV 600 mg/m^2^
Cycle length: 21 days Total cycles: 4 cycles	
Dose-dense AC-T (AC, doxorubicin and cyclophosphamide; T, paclitaxel)	
Doxorubicin	IV 60 mg/m^2^
Cyclophosphamide	IV 600 mg/m^2^
Cycle length: 14 days Total cycles: 4 cycles	
And	
Paclitaxel	IV 175 mg/m^2^
Cycle length: 14 days Total cycles: 4 cycles	
Or	
Paclitaxel	IV 80 mg/m^2^
Cycle length: 7 days Total cycles: 12 cycles	
TCH (docetaxel, carboplatin, and trastuzumab)^a^	
Docetaxel	IV 75 mg/m^2^
Carboplatin	IV AUC for 6 mg/mL/minute
Cycle length: 21 days Total cycles: 6 cycles	
AC-TC (AC, doxorubicin and cyclophosphamide; TC, paclitaxel and carboplatin)	
Doxorubicin	IV 60 mg/m^2^
Cyclophosphamide	IV 600 mg/m^2^
Cycle length: 14 days Total cycles: 4 cycles	
And	
Paclitaxel	IV 80 mg/m^2^
Carboplatin	IV AUC for 6 mg/mL/minute

This schema was used to determine if chemotherapy doses were reduced or delayed if not otherwise specified in clinician notes. Information was from UpToDate.^22^

^a^Trastuzumab was not included in our analysis.

**Table A2.** Chemotherapy regimen modifications by race.

	TC, *N* (%)		TCH, *N* (%)		AC-T, *N* (%)		AC-TC, *N* (%)		Other, *N* (%)	
	White (*N* = 73)	Black (*N* = 14)	White (*N* = 31)	Black (*N* = 13)	White (*N* = 60)	Black (*N* = 20)	White (*N* = 19)	Black (*N* = 2)	White (*N* = 25)	Black (*N* = 13)
Dose delayed										
Initiation delayed	7 (10%)	2 (14%)	2 (6%)	1 (8%)	2 (3%)	0 (0%)	0 (0%)	1 (50%)	3 (12%)	1 (8%)
Delay during chemotherapy	12 (16%)	3 (21%)	7 (23%)	7 (53%)	30 (50%)	7 (35%)	15 (79%)	1 (50%)	9 (36%)^a^	11 (85%)^a^
Dose reduction	12 (16%)	1 (7%)	16 (52%)^a^	12 (92%)^a^	20 (33%)	9 (45%)	14 (74%)	1 (50%)	9 (36%)	8 (62%)
Early treatment discontinuation	6 (8%)	3 (21%)	6 (19%)	1 (8%)	10 (17%)^a^	9 (45%)^a^	7 (37%)	1 (50%)	10 (40%)^a^	10 (77%)^a^
Hospitalization	9 (12%)	1 (7%)	5 (16%)	4 (31%)	16 (27%)	6 (30%)	3 (16%)	1 (50%)	5 (20%)	4 (31%)

TC, docetaxel/cyclophosphamide (±anti-HER-2); TCH, docetaxel/carboplatin/anti-HER-2; AC-T, doxorubicin/cyclophosphamide before/after paclitaxel; AC-TC, doxorubicin/cyclophosphamide before/after paclitaxel/carboplatin.

^a^Statistically significant *P*-value (*P* < .05).

**Table A3.** Unadjusted analysis of predictors with RDI <85% for at least one agent.

Predictor	Total *N* (*N* = 207)	Patients experiencing RDI <85%, *N* (%)	RR (95% CI)
Age			
<65	143	42 (29%)	1.00
≥65	64	14 (22%)	0.74 (0.44-1.26)
Race			
White	163	40 (25%)	1.00
Black	44	16 (36%)	1.48 (0.92-2.38)
Stage			
1	43	12 (28%)	1.00
2	111	25 (23%)	0.81 (0.45-1.46)
3	53	19 (36%)	1.28 (0.70-2.34)
Chemotherapy timing			
Neoadjuvant	80	24 (30%)	1.00
Adjuvant	127	32 (25%)	0.84 (0.54-1.32)
Anthracycline-based regimen			
No	114	26 (23%)	1.00
Yes	93	30 (32%)	1.41 (0.90-2.21)
